# Digital Droplet Multiple Displacement Amplification (ddMDA) for Whole Genome Sequencing of Limited DNA Samples

**DOI:** 10.1371/journal.pone.0153699

**Published:** 2016-05-04

**Authors:** Minsoung Rhee, Yooli K. Light, Robert J. Meagher, Anup K. Singh

**Affiliations:** Sandia National Laboratories, Biotechnology and Bioengineering Department, Livermore, California, United States of America; University of Michigan, UNITED STATES

## Abstract

Multiple displacement amplification (MDA) is a widely used technique for amplification of DNA from samples containing limited amounts of DNA (e.g., uncultivable microbes or clinical samples) before whole genome sequencing. Despite its advantages of high yield and fidelity, it suffers from high amplification bias and non-specific amplification when amplifying sub-nanogram of template DNA. Here, we present a microfluidic digital droplet MDA (ddMDA) technique where partitioning of the template DNA into thousands of sub-nanoliter droplets, each containing a small number of DNA fragments, greatly reduces the competition among DNA fragments for primers and polymerase thereby greatly reducing amplification bias. Consequently, the ddMDA approach enabled a more uniform coverage of amplification over the entire length of the genome, with significantly lower bias and non-specific amplification than conventional MDA. For a sample containing 0.1 pg/μL of E. coli DNA (equivalent of ~3/1000 of an *E*. *coli* genome per droplet), ddMDA achieves a 65-fold increase in coverage in de novo assembly, and more than 20-fold increase in specificity (percentage of reads mapping to E. coli) compared to the conventional tube MDA. ddMDA offers a powerful method useful for many applications including medical diagnostics, forensics, and environmental microbiology.

## Introduction

Whole genome sequencing is beneficial for the study of samples with limited DNA such as difficult-to-culture microorganisms and the analysis of clinical samples [1–6], but most DNA sequencing technologies require nanogram to microgram amounts of DNA for library preparation, while a single bacterial or human cell contains only a few femtograms or picograms of DNA template. When dealing with the limited amounts of DNA from a single or a few cells, it is necessary to perform whole genome amplification to obtain sufficient material for preparation of a sequencing library [7]. Multiple displacement amplification (MDA) is the most common of several techniques used [8–9] for amplifying limited input DNA [3]. MDA is an isothermal method using random primers and the strand-displacing ϕ29 DNA polymerase for high yield, high fidelity amplification [10–12]. The ϕ29 polymerase has an error rate at least one order of magnitude lower than other DNA polymerase enzymes, which is a major advantage of MDA for high fidelity genomic studies. MDA generates substantially more DNA than thermal cycling processes such as PCR [13]. Despite its advantages, MDA is hampered by amplification bias and non-specific amplification that may compromise subsequent genome sequencing [14–15]. when using less than a nanogram of starting template [16–17] Amplification bias is caused by preferential priming of certain sequences, which leads to highly uneven representation of the template DNA [6, 18] after exponential amplification. Nonspecific amplification is caused by exogenous DNA present in the reagents, or by formation of amplifiable dimers of the random primers used for MDA [10, 19]. The first demonstration of single cell genome sequencing using MDA reported >10^9^ fold amplification of isolated *E*. *coli* single cells (~5 fg) [20]. However, only 30% of the DNA amplicon was specific to the original *E*. *coli* sequence, and 70% was derived from random primer dimers or other DNA contaminants.

One way to improve MDA amplification for small amounts of template is to use smaller reaction volumes to increase the effective template DNA concentration while maintaining the same concentrations of other reagents, including any contaminating DNA. For example, Hutchison *et al*. demonstrated that improved specificity could be achieved by reducing the volume of the MDA reaction from 50 μL to 600 nL, although no clear improvement was observed in amplification bias [21]. Marcy *et al*. used a microfluidic device to further reduce the MDA reaction volume down to 60 nL. This increased the specificity up to 80–95% from a single cell and reduced sequencing bias as well [22]. Applying the same principle of volume reduction, digital MDA (dMDA) in an array of ~6 nL microfluidic chambers was used as a method to detect extremely small amounts of DNA fragments of unknown sequence [10]. More recently, MDA in microfabricated wells (~12 nL) was demonstrated, with a modified MDA protocol incorporating a second strand-displacing DNA polymerase [1]. They confirmed more than 80% of assembled bases were mapped to the original *E*. *coli* template and showed 88–94% coverage of the entire *E*. *coli* genome.

All of the techniques presented above to improve MDA performance require complex or labor-intensive protocols, or rely on complex, custom-fabricated microfluidic devices that constrain the reaction volume to nanoliter level. Here, we present droplet-based whole bacterial genome amplification in millions of sub-nanoliter droplets, which we call droplet digital MDA (ddMDA). The only custom equipment required is a microfluidic droplet generator, versions of which are available commercially. We demonstrate that simply partitioning a conventional MDA reaction into many droplets with volumes of 150 pL improves the quality of whole genome amplification, compared to an identical reaction performed at a conventional microliter scale.

## Materials and Methods

### Device fabrication and operation

The microfluidic droplet generators were built as previously described [23]. For the continuous phase, fluorinated oil (HFE-7500, 3M) with 2% (w/w) surfactant (PicoSurf®, Dolomite) was used. All fluids were actuated by pressurizing off-chip reservoirs using 0–15 psi scalable pressure modulators (Pneutronics) connected to in-house nitrogen gas. The typical range of operation was 1.9–2.1 psi. The pressure modulators were controlled by a custom LabView interface. Device operation was monitored using an Olympus IX71 inverted microscope equipped with an interline CCD camera (Andor Clara). Droplets were collected into a microcentrifuge tube for incubation immediately following droplet generation.

### MDA reactions

*Escherichia coli* K-12 MG1655 (ATCC, Manassas, VA) was cultured in Luria broth and the cell concentration was determined by optical density at 600 nm (OD_600_ of 1.0 = 1x 10^9^ cells/ml) after washing with PBS. The cells were diluted to four different concentrations in water and heated at 95°C for 10 minutes for thermal lysis and denaturation followed by immediate cool-down on ice. MDA reaction mixture consisted of 1X MDA reaction buffer (40 mM Tris-HCl (pH7.5), 50 mM KCl, 10 mM MgCl_2_, and 5 mM (NH4)_2_SO_4_), 0.5 mM dNTPs, 50 μM random hexamers, 4% PEG-400, 2% DMSO, 20 μM SYBR Green and 0.15 μM of *ϕ*29 DNA polymerase (New England Biolabs, Ipswich, MA). MDA ready samples were prepared by adding the MDA reaction mixture to varying concentrations of the thermally lysed cells. The final volume for the samples was 300 μL. A 20 μL aliquot from each concentration was taken for tube MDA and transferred to a 200 μL PCR tube, while the rest 280 μL was used for droplet MDA. The reaction mixes containing template and MDA mix were maintained at 4°C until they were immediately emulsified into droplets of 150 pL average volume using the droplet generation chip. Multiple replicates of 60 μL volume of MDA droplets (approximately consisting of 42 μL of aqueous droplets and 18 μL of fluorinated oil) were collected into 200 μL PCR tubes. The PCR tubes for tube MDA and ddMDA were brought together to the thermocycler (PTC-225 DNA Engine Thermocycler, MJ Research) and incubated under the same condition at 30°C for 18 hours. We used a standard MDA protocol without extraordinarily stringent cleaning steps [24–25] or post-amplification treatments for purification. We excluded the effect of the increased effective concentration of template DNA by using the same concentrations of all reagents for both tube MDA and ddMDA.

### Sequencing library preparation

The amplified DNA (60 μL of ddMDA or 20 μL of tube MDA) was cleaned using a DNA Clean & Concentrator™-5 (Zymo Research, Irvine, CA) and the control genomic DNA was prepared from 10ml of overnight culture of E.coli K-12 MG1655 using ZR Fungal/Bacterial DNA MiniPrep™ (Zymo Research) by following the manufactures’ protocols. After quantification using Qubit® dsDNA HS Assay Kit (Life Technologies, San Diego, CA), one nanogram each of purified DNA from the MDA samples and one nanogram of genomic DNA were prepared for a sequencing library using Nextera XT kit (Illumina, San Diego, CA) and pooled together as the manufacture’s protocol. Sixteen picomolar of the pooled library was loaded to MiSeq (Illumina) for a 75-cycle paired-end reads using the v3 chemistry.

### Mapping, mapping visualization and genome assembly

MiSeq output fastq sequences were filtered and trimmed using Trimmomatic [26]. For the mapping analysis, single-end reads were mapped to *E*. *coli* K-12 MG1655 reference genome (NCBI, NC_000913.3) using Bowtie 2 with default settings in ‘end-to-end’ alignment mode [27]. The mapping coverage to the genome was visualized by BRIG 0.95 using the SAM files from Bowtie 2 as input files [28]. GC content of the reference genome was calculated with sliding windows, 10000 bases for whole genome and 2000 bases for zoomed-in region, using Bioconductor packages and genome coverage was visualized with Sushi [29] using bedgraphs created by bedtools (v.2.24.0) genomecov suite [30] as input files. The *de novo* assembly of sequences was performed using SPAdes 3.5 genome assembler [31] with ‘single-cell’, ‘paired-end libraries’ and ‘careful’ options and was evaluated using QUAST [32]. Lorez curve and Gini Index were generated with read depths at each genome position obtained using bedtools (v.2.24.0) genomecov suite and binned using R.

## Results and Discussion

The amplification bias during conventional MDA is mostly caused by preferential priming, where certain preferred portions of the template are repeatedly favored and exponentially amplified as the reaction continues. This results in uneven representation of the template, and uneven coverage in sequencing [33]. Small amounts of template DNA in the sample also leads to increased non-specific amplification as chimeras and contaminating DNA represent a larger fraction of the total amplified DNA relative to the template.

**Fig 1:**
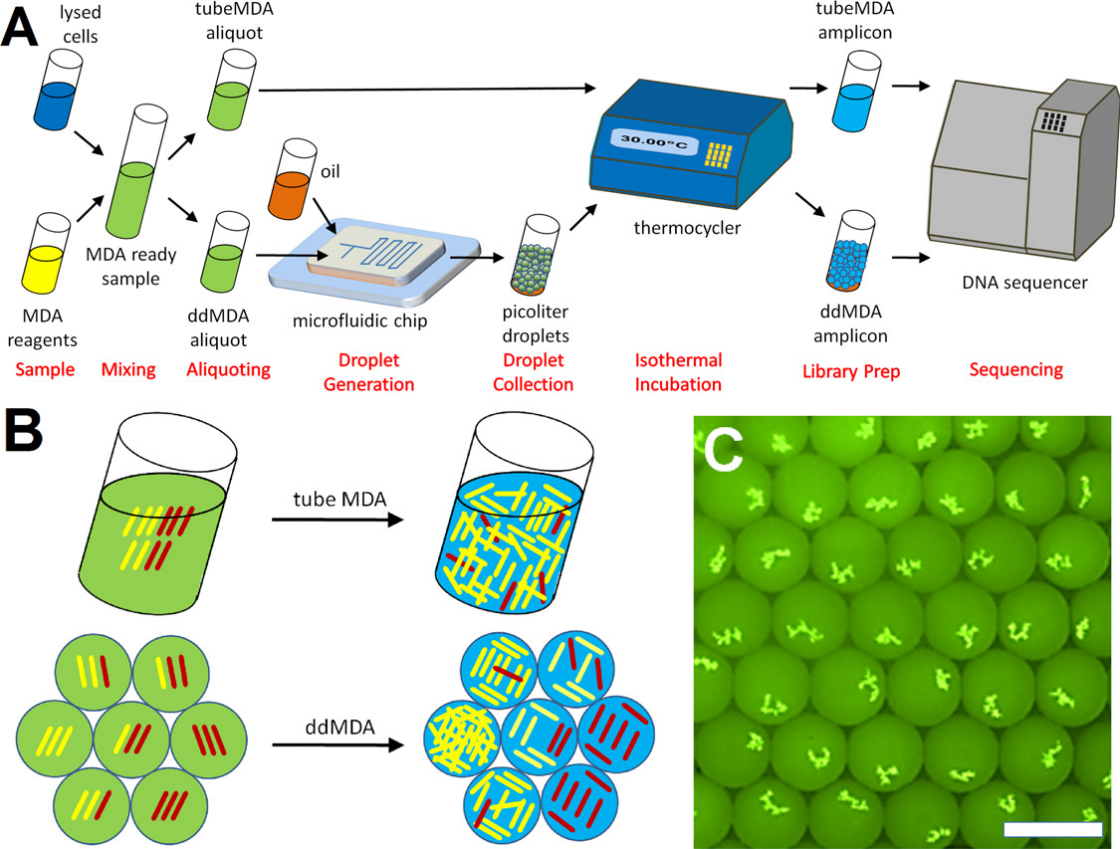
The working principles of ddMDA. (A) The ddMDA procedures as a high quality alternative to the conventional tube MDA. The MDA ready *E*. *coli* samples were partitioned into millions of picoliter droplets using a microfluidic droplet generator. Upon collection, droplets were tightly sealed for isothermal incubation at 30°C for 18 hours. DNA amplicons were then purified, cleaned, and prepared for the following sequencing. (B) Denatured and fragmented whole genomes consist of highly amplifiable (yellow) and weakly amplifiable (red) sequences. During tube MDA, yellow fragments are preferred and repeatedly amplified with a high gain until it reaches a concentration plateau, whereas red fragments are less preferred and barely amplify. For ddMDA, DNA fragments are randomly partitioned into picoliter droplets, resulting in different subsets of the template DNA. When a droplet contains yellow fragments, the amplification kinetics favor the yellow fragments, ending up with significant biases on amplification. The enzyme will amplify red fragments at a slower rate only in the absence of yellow fragments. The overall gain of ddMDA is always lower than tube MDA because of the volume constraint. Every droplet is uniquely composed of fragments and ends up with a different amplification gain after MDA. (C) A fluorescence micrograph showing ddMDA endpoint with the initial template DNA concentration of 100 pg/μL. Having started with different parts of the *E*. *coli* genome, individual droplets expressed discrete levels of amplification by showing different sizes of DNA amplicon aggregates and different fluorescent signals. The scale bar shows 100μm.

We address both the bias in amplification and non-specific amplification by performing MDA in droplets where a sample containing *E*. *coli* genomic DNA into millions of ~150 pL droplets which are amplified in parallel, and then pooled to generate a single sequencing library. This workflow is illustrated in Fig 1A. At the beginning of the reaction, each droplet contains a small subset of the total population of DNA fragments, and amplification in each droplet occurs independently from the other droplets. This reduces the effect of competition for preferential priming and access to polymerase, leading to more even amplification of fragments covering the entire genome (Fig 1B). Contaminating DNA fragments that are present in low initial concentration are also partitioned into a small number of droplets, limiting the representation of these sequences in the final pooled library. Fig 1C shows a fluorescence micrograph of the end product of ddMDA reaction, with an array of droplets each of which contains a discrete hyper-branched MDA product.

Another feature of the ddMDA reactions was identified is the decreased amplification gain. It is likely that the volume restriction in each droplet limits the amplification reaction and decreases the gain. However, the reduction in amplification gain eventually prevents unlimited exponential growth of preferred sequences, resulting in improved coverage and uniformity of amplification. The amplification gain is thus not only an absolute measure of the amplification yield, but it is also an indirect indicator of the quality of whole genome amplification [34], where large gains generally correlates with increased amplification bias. It has been known that gains greater than 10^7^ significantly deteriorate amplification chemistry, resulting in poor de novo assembly [7]. This observation suggests that it the amplification gain should be minimized to the level that is barely sufficient for the subsequent sequencing. Average gains for different initial DNA concentrations are shown in Fig A in S1 File. Depending on the initial DNA concentrations, our ddMDA yielded 10^1^−10^5^ gains of amplification, which was far lower than the typical gains in tube MDA (>10^7^) which are associated with poor quality amplification libraries. The amplification gain indeed gradually increased as the volume of droplets increased, which justified the small volume constraints on the gain (Fig B in S1 File). The sacrificed yields from the reduced gain of ddMDA can be compensated with an enormously large number of MDA droplets that enables ultra-high throughput. For sequencing purposes, we collected ~280,000 ddMDA droplets (~ 42 μL in total volume) for each run.

### Reduction of amplification bias

**Fig 2:**
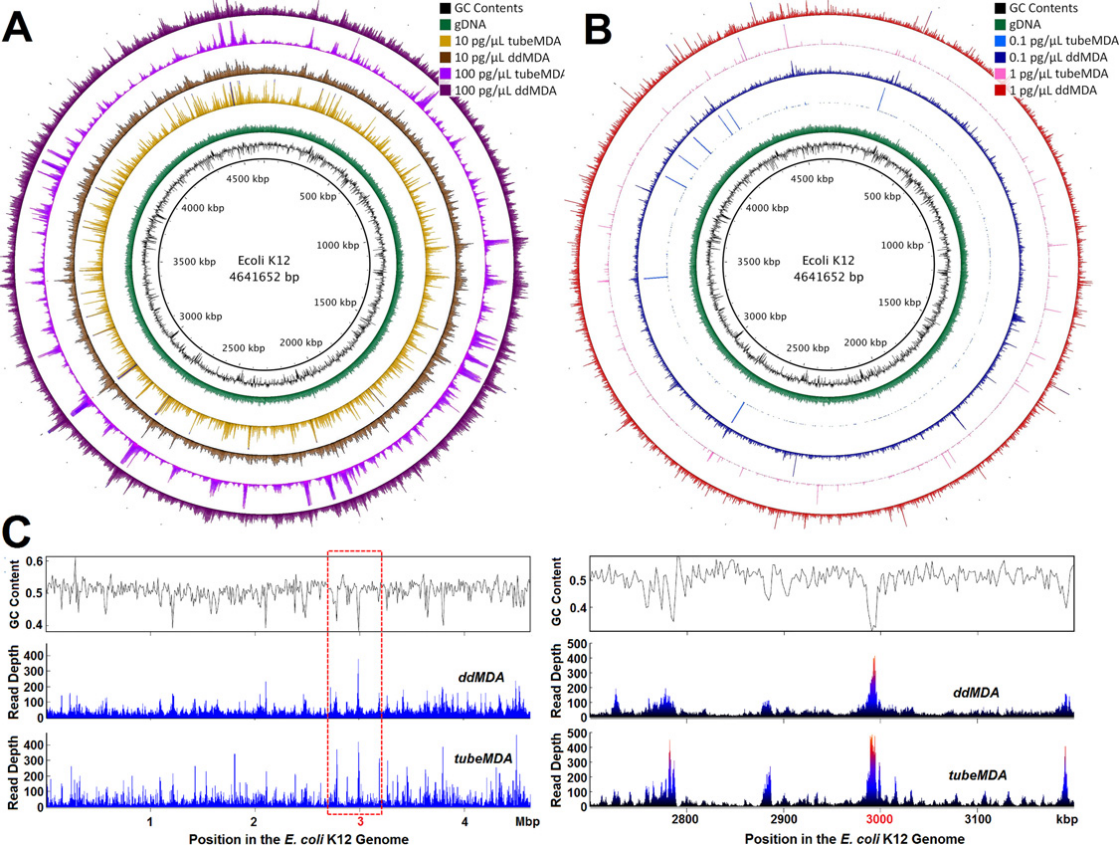
Comparison of whole genome coverage of assembled contigs mapped onto *E*. *coli* K12 genome sequences for ddMDA and tube MDA. (A) From the outermost circle, ddMDA for 100 pg/μL (dark purple), tube MDA for 100 pg/μL (bright purple), 10 pg/μL (dark brown), and 10 pg/μL (bright brown), respectively. GC contents across the genome were depicted in black in the innermost circle and reads from genomic DNA were illustrated in green as a reference. (B) From the outermost circle, ddMDA for 1 pg/μL (dark red), tube MDA for 1 pg/μL (bright red), 0.1 pg/μL (dark blue), and 0.1 pg/μL (bright blue), respectively. (C) GC contents (top) and amplification read depths over the entire E. coli genome for ddMDA (middle) and tube MDA (bottom) at the DNA concentration of 10 pg/μL. The right panels show zoomed-in plots of the dotted-line box region of the genome for close-up visualization.

To test whether ddMDA alleviates amplification bias, we amplified *E*. *coli* genomes by performing tube MDA (20 μL total volume) and ddMDA (42 μL total volume, partitioned into ~300,000 droplets of 150 pL volume), with template DNA concentrations varying from 0.1 pg/μL to 100 pg/μL. We then sequenced the same mass (1 ng) of amplicon prepared from each method. As a control, an amplification-free sequencing library was also prepared directly from 1 ng of *E*. *coli* genomic DNA. Fig 2A and 2B shows genome coverage and read depths from tube and droplet MDA with various initial DNA concentrations. Both the coverage and the uniformity of read depths deteriorate over the entire genome as the template concentration decreased from 100 pg/μL to 0.1 pg/μL. However, ddMDA had a markedly improved quality of amplification compared to tube MDA at the same concentrations, and this effect is most pronounced at low template DNA concentrations (Fig 2B).

In the coverage maps, empty disconnected regions show missing sequences of the genome where MDA completely failed to amplify DNA. Peaks and valleys reflect regions with high bias in amplification. Most of the coverage peaks appeared at the same positions regardless of the reaction volume and DNA concentrations. This implies that the preferential bias during MDA is inherent and systematic. Furthermore our results are consistent with previous observations that bias is correlated to GC content of template DNA [35–36]. This is illustrated in Fig 2C, with zoomed-in plots for extreme GC content regions of an *E*. *coli* genome showing explicit amplification peaks around GC poor regions and valleys around GC rich regions. Note that tube MDA was more sensitive to GC content while ddMDA produced more uniform amplification over the entire genome. While our data is most suggestive of a bias against GC-rich sequences, amplification bias is likely a complex phenomenon arising from multiple parameters such as repetitive regions, template size, GC content, method of denaturation, incubation temperature, and concentration of reactants.

Early literature suggested that pooling of replicate MDA reactions would substantially relieve the amplification bias by making the coverage depth average out statistically [37], but this was subsequently disproven [38]. Since bias is systematic, pooling of identical microliter-scale MDA reactions with the same template DNA does not improve amplification coverage. By contrast, ddMDA uses hundreds of thousands of droplets, each with a very few fragments comprising different subsets of the total template DNA. Although we ultimately pool the droplets to prepare a single sequencing library, the droplet partitioning is different from pooling a small number of identical microliter-volume reactions.

### Improvement of specificity in ddMDA

**Fig 3:**
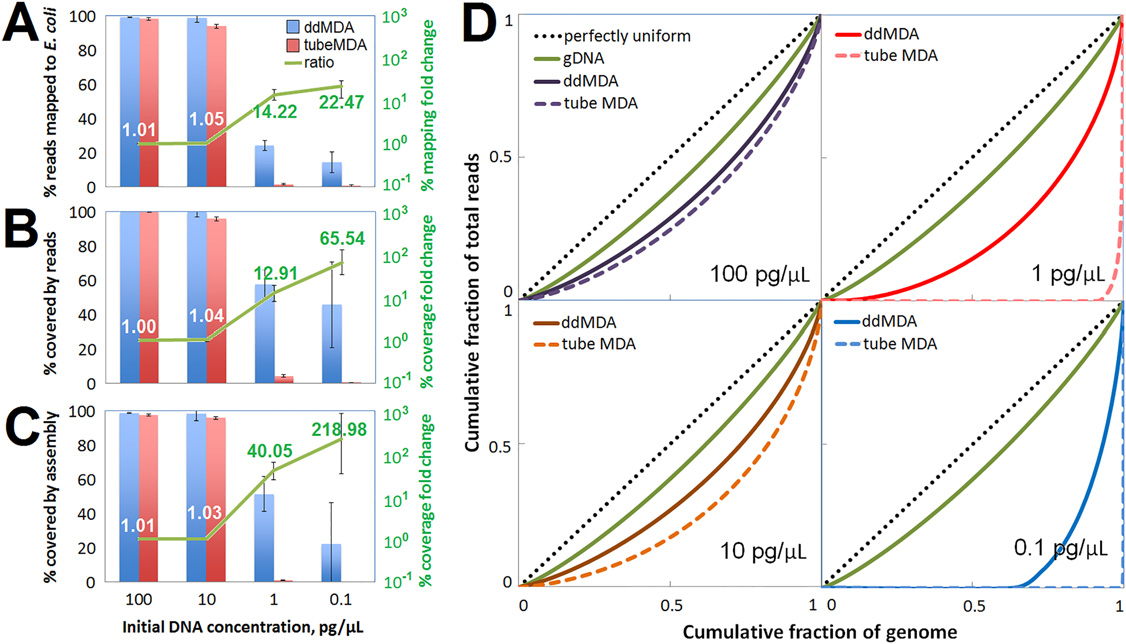
Improvement of the overall quality of amplification by ddMDA. (A) The fraction of reads correctly mapped to *E*. *coli* genome depending on the DNA concentration and the MDA reaction volume. Blue and red columns show ddMDA and tube MDA results, respectively. The green line shows the fold change of the % mapping from tube MDA to ddMDA (= ddMDA/tubeMDA). It stayed around 1 for high concentrations (10–100 pg/μL) while it considerably increased for low concentrations (0.1–1 pg/μL). (B) The fraction of an *E*. *coli* genome covered by one or more sequencing reads depending on the DNA concentration and the MDA reaction volume. Tube MDA and ddMDA showed little difference at high concentrations but the fold change significantly increased at low concentrations. (C) The fraction of an *E*. *coli* genome covered by contigs during de novo assembly. While the advantage of ddMDA over tube MDA was still limited at high concentrations but the fold change significantly increased at low concentrations. (D) Lorenz curves depict the amplification bias in read coverage across the *E*. *coli* genome. Each curve was calculated by evaluating the read depth for each base and using the resultant cumulative distribution function for read depth to determine the cumulative proportion of total genome coverage (y-axis) accounted for by the cumulative proportion of bases (x-axis). The ideal Lorentz curve (black dotted line) for a distribution in which all of the bases have the same coverage and a Lorenz curve for gDNA were plotted for comparison. Other solid curves show ddMDA curves while dotted curves indicate tube MDA. (Upper Left) For 100 pg/μL, ddMDA in dark purple and tube MDA in bright purple. (Bottom Left) For 10 pg/μL, ddMDA in dark brown and tube MDA in bright brown. (Upper Right) For 1 pg/μL, ddMDA in dark red and tube MDA in bright red. (Bottom Right) For 0.1 pg/μL, ddMDA in dark blue and tube MDA in bright blue.

In an MDA reaction with limited template, small amounts of exogenous or contaminating DNA behaves similarly to highly amplified product and can outcompete template for amplification. In ddMDA, we reduce the impact of contaminating DNA by partitioning template into droplets, which limits the extent to which it can compete with template DNA. The specificity of amplification is determined from the fraction of the total sequencing reads that mapped to the *E*. *coli* genome (Table 1 and Fig 3A). We found that, at low concentrations of template DNA, the specificity greatly increased in ddMDA compared to tube MDA. Although ddMDA and tube MDA were not significantly different for high concentrations of template (1–10 pg/μL), our ddMDA showed 14-fold higher fraction of reads mapping to *E*. *coli* than the tube MDA for 1 pg/μL template, and 22.5-fold higher mapping at 0.1 pg/μL template.

**Table 1:** Statistics of sequence mapping and assembly of *E*. *coli* K12 MG1655 samples prepared with ddMDA and conventional tube MDA at different initial copy numbers. Total number of reads included all sequencing reads mapped and unmapped to *E*. *coli* genome. % Reads mapped to genome corresponds to the percentage of sequencing reads that are specifically aligned to *E*. *coli* genome. % Genome covered by reads refers to the percentage of *E*. *coli* genome covered by one or more sequencing reads. % Genome covered by assembly indicated the percentage of *E*. *coli* genome covered by contigs. Gini indices were calculated based on the cumulative distribution of sequencing reads.

Sample/Method	Initial Concentration (pg/μL)	Total # of Reads	Total (bp)	% Reads Mapped to Genome	% Genome Covered By Reads	% Genome Covered by Assembly	Gini Index
gDNA	-	2185284	4564369	99.75	99.89	98.40	0.17
tube MDA	100	3896826	4569435	98.09	99.76	97.46	0.37
tube MDA	10	1311506	4451316	93.77	95.77	95.79	0.49
tube MDA	1	2149455	68031	1.71	4.47	1.28	0.98
tube MDA	0.1	1837463	4777	0.64	0.70	0.10	1.00
ddDMA	100	1477081	4573241	98.88	99.99	98.70	0.31
ddDMA	10	1236961	4572794	98.46	99.97	98.42	0.34
ddDMA	1	1930972	2536900	24.30	57.19	51.39	0.53
ddDMA	0.1	1385553	1128740	14.38	28.38	22.12	0.81

### De novo assembly of ddMDA products

Quantitative metrics of sequencing coverage and bias are presented in Fig 3 and Table 1. As shown in Fig 3B, the sequencing coverage substantially improved as the concentration increased, which is expected. At high template concentrations (10–100 pg/μL), ddMDA and tube MDA showed almost the same level of coverage at high concentrations. However, ddMDA enabled approximately 13-fold higher coverage at 1 pg/μL and 65-fold higher coverage at 0.1 pg/μL than tube MDA.

Sequence data from both tube MDA and ddMDA libraries were used for *de novo* assembly of the *E*. *coli* genome. We assembled >98% of the *E*. *coli* genome from ddMDA when the starting template concentrations were 10 pg/μL and higher (Table 1, Fig 3C), with N50 contig sizes longer than 132 kbp and maximum contig lengths of >268 kbp (Table A in S1 File). The high assembly fraction and the long contig lengths indicate successful high quality amplification. Tube MDA with the same initial concentrations yielded slightly lower coverage levels (95.8–97.5%), which means that the partitioning offered only marginal improvement at such high concentrations. The effect of ddMDA is more dramatic at lower template concentrations, where the assembled coverages for ddMDA showed a 40–220 fold increase compared to those of tube MDA, and a larger number of contigs greater than 500 bases.

The Lorenz curve can be used for read depth distributions of sequencing results to represent uniformity of the sequencing read distribution^7^. A perfectly uniform read distribution would be one in which every base has the same number of reads, and would be plotted as the straight line y = x (line of perfect uniformity of reads). By contrast, a perfectly unequal distribution would be one in which one base has all the reads and all other bases have none. In that case, the curve would be at y = 0 for all x < 1, and y = 1 when x = 1. Fig 3D illustrates Lorenz curves for sequencing read distributions for different MDA methods with various DNA concentrations. Consistent with Fig 2, higher initial DNA concentrations showed more uniform distributions and our ddMDA yielded greater uniformity than tube MDA for all concentrations although the effect was more significant for lower initial DNA concentrations. The uniformity based on Lorenz curve analysis can be numerically represented as a Gini index (or a Gini coefficient). A perfectly uniform distribution gives a Gini index of 0 while a perfectly unequal distribution gives 1. Gini indices of the read distributions from our ddMDA and tube MDA experiments are listed in Table 1. At all concentrations, ddMDA results in a lower Gini index (meaning higher uniformity of coverage), and this becomes most pronounced at the lower template concentrations (1 pg/μL and 0.1 pg/μL). At these concentrations, tube MDA shows highly biased amplification with Gini coefficients approaching 1. The ddMDA Gini coefficients, while still showing evidence of uneven coverage, are significantly lower than 1, at 0.53 and 0.81.

## Conclusions

We showed that performing ddMDA in water-in-oil droplets generated with a simple microfluidic device substantially improved the quality of whole genome amplification compared to a conventional MDA reaction, which was attributed to discretization of template DNA by partitioning into numerous small reaction volumes. The amplification gain depended upon the initial DNA concentration in droplets, and was overall lower than tube MDA where high gain is correlated with high bias. We applied ddMDA to single *E*. *coli* cells and accomplished sequencing of almost the whole genome. From *de novo* assembly, we found that >98% of the genome was correctly assembled via ddMDA at 10 pg/μL or higher concentrations, and significantly higher coverages were achieved than tube MDA at lower template concentrations as well. While tube MDA showed extreme deterioration of amplification quality for initial DNA concentrations of 1 pg/μL (~200 *E*. *coli* genomes/μL) or lower, our ddMDA significantly reduced the lower limit of initial DNA concentration for reliable amplification down to 0.1 pg/μL, which corresponds to ~20 *E*. *coli* genomes/μL, or only ~0.3% of an *E*. *coli* genome per droplet. Note that these results were achieved without using any of the stringent precautions that are often taken for low-template MDA, such as UV irradiation of all reagents or operation in a specialized clean environment. In summary, the benefits of using droplets with picoliter reaction volumes for MDA include improved specificity of amplification, reduction in amplification bias, and highly improved amplification coverage, which are attributed solely to partitioning the template into a large number of small reaction volumes. A most recent publication [39] showed a good agreement with our findings; yet our study further extended to explain the rationale behind the sequencing quality improvement by showing the preferential bias change depending on GC content and differential amplification gain in droplets.

These results suggest that applying ddMDA to DNA from unculturable organisms would increase the diversity of species amenable to genomic study. The improved specificity and coverage of ddMDA justifies its widespread use in sequencing clinical and environmental samples with limited amounts of DNA, so that novel genomes, chromosomes, genes, and viruses can be amplified and characterized in a high-throughput manner. We note one recent study that demonstrates an emulsion-based MDA technique improves performance with DNA derived from small amounts of human DNA, including high-accuracy detection of SNPs and copy number variation [40]. Coupling ddMDA with on-demand droplet techniques [41] or droplet sorting methods [42] would further increase its potentials in metagenomics as well. The current ddMDA protocol leaves room for improvement by employing stringent sample preparation for complete elimination of exogenous DNA, which would further enhance the specificity of amplification. We are also exploring integration of droplet generation, denaturation and amplification into a microfluidic device, although this requires at least one reagent addition step which could be accomplished by picoinjection or droplet merging [23]. Topics for further study and optimization include the effect of denaturation technique (the thermal denaturation used here, versus alkaline denaturation which is more common), the effect of template fragment size prior to partitioning, and the optimal number and volume of droplets for a given amount and complexity of template DNA.

## Supporting Information

S1 File Discretization of amplification by ddMDA; Skewness of sequencing reads depth distribution. Table A. Supplementary statistics of sequencing assembly. Table B. Supplementary statistics for determination of skewness of a distribution. Fig A. Average amplification gains and final concentrations of DNA amplicons depending on the initial DNA concentration. Fig B. The change of amplification gains at different initial DNA concentrations for ddMDA as a function of the reaction volume (= the volume of droplets). Fig C. Comparison of sequencing results over the whole *E*. *coli* genome between replicate runs for ddMDA and tube MDA. Fig D. Comparison of statistical representative metrics to determine the degree of skewness of the read depth distributions.
(DOCX)
