# Supplementary material for: Digital Droplet Multiple Displacement Amplification (ddMDA) for Whole Genome Sequencing of Limited DNA Samples
Source: PLoS One. 2016 May 4;11(5):e0153699. doi: 10.1371/journal.pone.0153699 (PMC4856258; doi:10.1371/journal.pone.0153699)
Supplement: S1 File — Table A. Supplementary statistics of sequencing assembly. Table B. Supplementary statistics for determination of skewness of a distribution. Fig A. Average amplification gains and final concentrations of DNA amplicons depending on the initial DNA concentration. Fig B. The change of amplification gains at different initial DNA concentrations for ddMDA as a function of the reaction volume (= the volume of droplets). Fig C. Comparison of sequencing results over the whole E. coli genome between replicate runs for ddMDA and tube MDA. Fig D. Comparison of statistical representative metrics to determine the degree of skewness of the read depth distributions. (DOCX) [file pone.0153699.s001.docx]

**Supporting Information**

Digital Droplet Multiple Displacement Amplification (ddMDA) for whole genome sequencing of limited DNA samples

*Minsoung Rhee, Yooli K. Light, Robert J. Meagher^,*^ and Anup K. Singh^*^*

Sandia National Laboratories, Biotechnology and Bioengineering Department, Livermore, CA, USA

*Corresponding Authors

**Discretization of amplification by ddMDA**

Previous researches on small-volume MDA focused only on the increase of effective concentrations of template DNA by the reduction of volume, which led to higher specificity of amplification but had almost no impact on the amplification bias. The droplet technology is perfectly suitable for increasing the effective concentrations of dilute samples either by abandoning empty droplets or making template-encapsulated droplets on demand [1]. This concentration effect will be stronger for more dilute samples or as we further reduce the size of droplets. We, however, tried to minimize the effect of increased effective concentration for this work by maintaining the same concentrations for both tube and ddMDA. The key advantage of ddMDA, over traditional as well as small volume reactions, is the discretization of amplification reactions enabled by partitioning template DNA fragments into a very large array of droplets. During denaturation, genomes are fragmented into many long DNA sequences with different amplification sensitivity and preferences. In ddMDA, those individual fragments are independently encapsulated in discrete droplets, resulting in a wide distribution of compositions such that each droplet potentially has a small number of different subset of DNA fragments. If a droplet contains a highly amplifiable fragment, that sequences will be exponentially and almost exclusively amplified, as it would in tube MDA. In other droplets, however, if a highly amplifiable sequence is not present, the weakly amplifiable sequences that would otherwise have been outcompeted, are able to amplify, albeit more slowly than the highly amplifiable sequences. We hypothesize that, at low input DNA concentrations, bias is much reduced in ddMDA because each droplet with a unique composition of the genome amplifies to a plateau independently of the others. Subsequently pooling the library from ddMDA results in a more even representation of both the highly amplifiable and weakly amplifiable sequences.

**Skewness of sequencing reads depth distribution**

Highly uniform amplification leads to fairly symmetric read depth distributions showing that the mean, median, and mode of the distribution are very close to each other. When the preferential bias during MDA undermines amplification uniformity, the read depth distributions however become skewed, making the three metrics markedly different where the median and mode are much smaller than the mean. For severely-skewed distributions, the median and mode may be both zero, indicating that a majority of bases in the genome had no amplification and ended up with zero reads. It is also quite common to have the right tail of the distribution considerably longer relative to the left tail. Comparing the three metrics and their closeness should provide a straightforward means to analyze the skewness of the read depth distribution, which in turn gives information on amplification uniformity (Table B and Fig. D). Another widely-used indicator of skewness is Pearson’s moment coefficient of skewness. It would be zero when the distribution were perfectly symmetric, but in reality it shows non-zero values in either the positive (right-skewed) or the negative (left-skewed) direction while farther from zero when the distribution is more skewed. The reference gDNA sample showed the moment coefficient of skewness of 0.44 only, demonstrating that the gDNA sample consists of genome fragments with highly uniform fractions. For tube MDA, we noticed that the skewness rapidly increased from 2.64 (100 pg/µL) and 3.90 (10 pg/µL) to 43.05 (1 pg/µL) and 91.20 (0.1 pg/µL) as the initial concentration decreased. For ddMDA, however, the change of skewness upon the concentration was much slower, and at the same time, their moment coefficients of skewness were much lower than tube MDA with the same concentrations, showing only 2.02 (100 pg/µL) and 2.84 (10 pg/µL) to 7.49 (1 pg/µL) and 8.03 (0.1 pg/µL).

**References**

1. Rhee, M., Liu, P., Meagher, R.J., Light, Y.K., Singh, A.K. *Biomicrofluidics* **2014,** *8*, 034112.

Table A. Supplementary statistics of sequencing assembly

Table B. Supplementary statistics for determination of skewness of a distribution. Means, medians, and modes represented the number of reads per base. The Pearson moment coefficient was calculated as follows. $\gamma=E\left( \left( \left( X-\mu\right)/\sigma\right)^{3} \right)$ where $\mu$ is the mean and $\sigma$ is the standard deviation of the distribution.

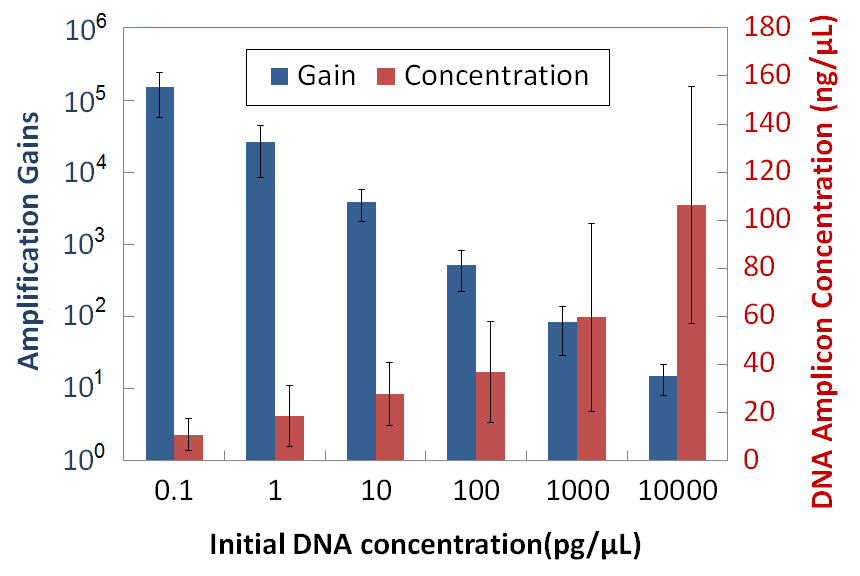


Fig. A. Average amplification gains (blue bars) and final concentrations of DNA amplicons (red bars) depending on the initial DNA concentration. Individual droplets were categorized into six concentration groups.


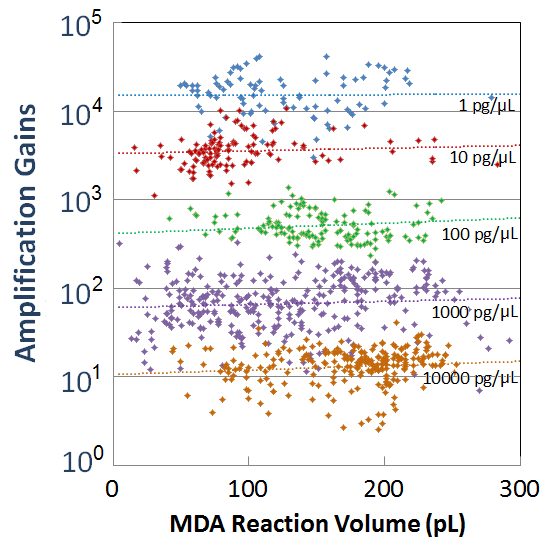


Fig. B. The change of amplification gains at different initial DNA concentrations for ddMDA as a function of the reaction volume (=the volume of droplets).


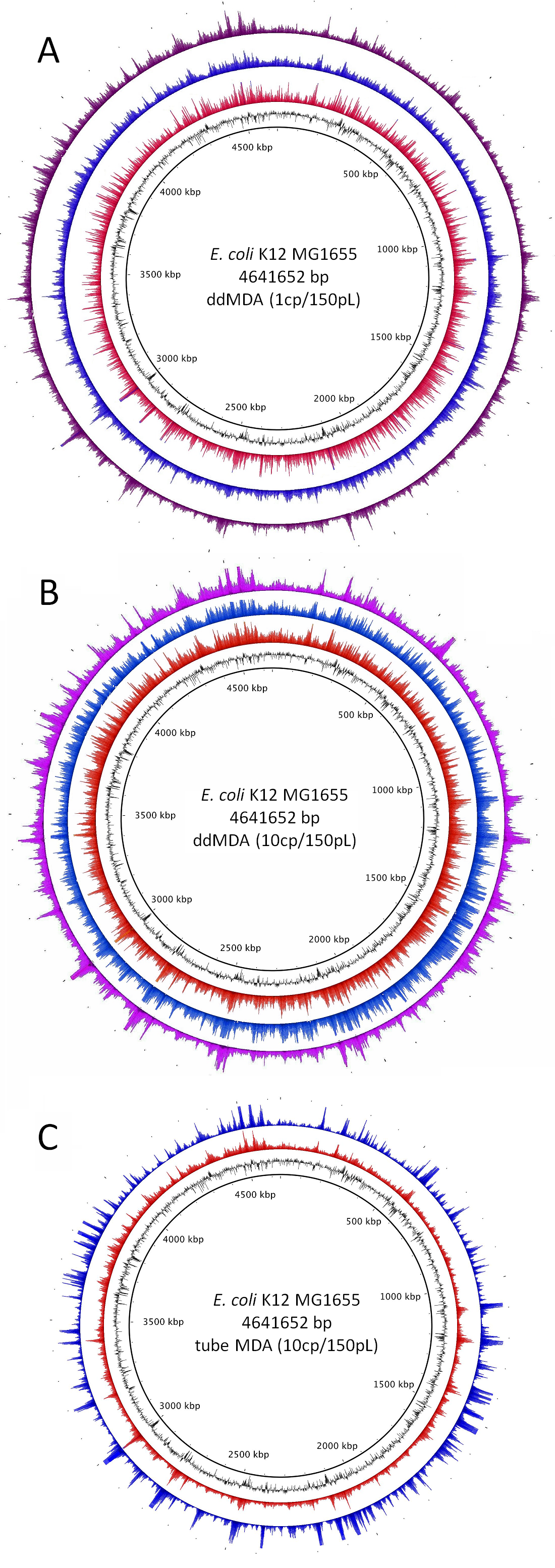


Fig. C. Comparison of sequencing results over the whole *E. coli* genome between replicate runs for A) ddMDA at 10 pg/µL, B) ddMDA at 100 pg/µL and C) tube MDA at 100 pg/µL. Note that peaks and valleys appear at the same locations around low GC content regions.


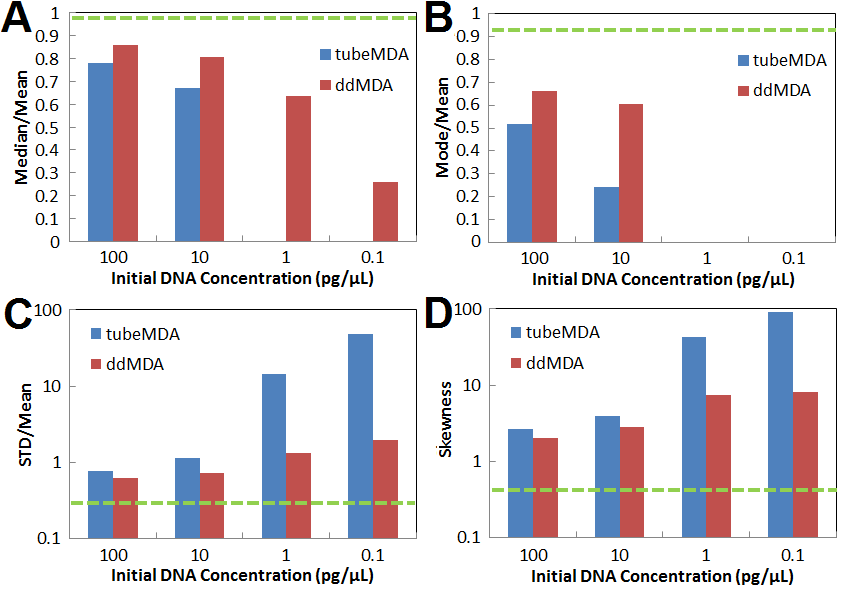
Fig. D. Comparison of statistical representative metrics to determine the degree of skewness of the read depth distributions. The green dotted line in each plot represents the value obtained with the reference gDNA. (A) Position of the median relative to the mean. Small values indicate high skewness. (B) Position of the mode relative to the mean. Small values indicate high skewness. (C) Standard deviation divided by the mean, which is reciprocal to the coefficient of variance. Large values indicate high skewness. (D) Pearson’s moment coefficient of skewness. Large values indicate high skewness.
